# Dietary N-Carbamylglutamate Supplementation Boosts Intestinal Mucosal Immunity in *Escherichia coli* Challenged Piglets

**DOI:** 10.1371/journal.pone.0066280

**Published:** 2013-06-19

**Authors:** Fengrui Zhang, Xiangfang Zeng, Fengjuan Yang, Zhimin Huang, Hong Liu, Xi Ma, Shiyan Qiao

**Affiliations:** State Key Laboratory of Animal Nutrition, Ministry of Agriculture Feed Industry Centre, China Agricultural University, Beijing, China; Charité, Campus Benjamin Franklin, Germany

## Abstract

N-carbamylglutamate (NCG) has been shown to enhance performance in neonatal piglets. However, few studies have demonstrated the effect of NCG on the intestinal mucosal barrier. This study was conducted to determine the effects of dietary NCG supplementation on intestinal mucosal immunity in neonatal piglets after an Escherichia coli (E. coli) challenge. New-born piglets (4 d old) were assigned randomly to one of four treatments (n = 7), including (I) sham challenge, (II) sham challenge +50 mg/kg NCG, (III) E. coli challenge, and (IV) E. coli challenge +50 mg/kg NCG. On d 8, pigs in the E. coli challenge groups (III and IV) were orally challenged with 5 mL of E. coli K88 (10^8^ CFU/mL), whereas pigs in the sham challenge groups (I and II) were orally dosed with an equal volume of water. On d 13, all piglets were sacrificed, and samples were collected and examined. The results show that average daily gain in the E. coli challenged piglets (III and IV) was decreased (P_E.coli_<0.05). However, it tended to be higher in the NCG treated piglets (II and IV). Ileum secretory IgA, as well as IFN-γ, IL-2, IL-4 and IL-10 in ileal homogenates, were increased in E. coli challenged piglets (III and IV). Similarly, ileum SIgA and IL-10 levels, and CD4^+^ percentage in NCG treated piglets (II and IV) were higher than no-NCG treated piglets (P_NCG_<0.05). However, the IL-2 level was only decreased in the piglets of E. coli challenge + NCG group (IV) compared with E. coli challenge group (III) (P<0.05). No change in the IL-2 level of the sham challenged piglets (III) was observed. In conclusion, dietary NCG supplementation has some beneficial effects on intestinal mucosal immunity in E. coli challenged piglets, which might be associated with stimulated lymphocyte proliferation and cytokine synthesis. Our findings have an important implication that NCG may be used to reduce diarrhea in neonatal piglets.

## Introduction

In modern pig farming, an increase in average litter size may enhance the potential for mortality from starvation and lack of innate immunity [1]. Hence, the development of immune system of neonatal piglets is particularly important. However, it is often underdeveloped [2]. For example, immunoglobulin quantitation, including IgA, IgG, and IgM, in the serum of young pigs decreased significantly at 14-d-old [3], and this may be due to an immature immune system, which is a main risk factor for infectious diseases in early life, especially the intestinal mucosal immunity [4].

It is well known that the intestine is the main entry route for foreign antigens, including invading pathogens that often lead to severe diarrhea [5]. Diarrhea in newborn piglets is a complicated problem caused by a variety of reasons, such as infectious agents like *E. coli* and rotavirus in small intestine [5], [6]. Neonatal piglet diarrhea often leads to a significant decline in body weight gain. A well developed intestinal mucosal immune system can protect the mucous membranes against potentially dangerous microbes and some other toxic elements in the environment [4]. Thus, many attempts to explore strategies to improve intestinal mucosal immunity and to understand the corresponding mechanisms have been made [7], [8].

Arginine, a nutritionally essential amino acid in young mammals, has attracted much interest because of its powerful physiologic properties and pharmacological role in intestinal mucosa [9]. It has been reported that dietary arginine supplementation can enhance immune response in different rat models [7], [10], improve intestinal function in weaned pigs [8], and stimulate mucosa growth in newborn piglets [11]. However, for milk-fed neonatal piglets, accumulated research indicates that arginine in sow’s milk cannot satisfy the requirement for piglets [12]. Meanwhile, the endogenous synthesis of arginine reduces dramatically in sucking piglets [13] owing to the decreasing activity of mitochondrial N-acetylglutamate synthase (NAGS) [9].

N-carbamylglutamate (NCG), a metabolically stable analogue of N-acetylglutamate (NAG), has been proved to increase the endogenous synthesis of arginine and plasma concentration of arginine by activating intestinal pyrroline-5-carboxylate synthase and carbamylphosphate synthase-1 [9]. Recent studies have proved that NCG supplementation could increase the serum arginine level, enhance pregnancy outcome in rats [14], and increase muscle protein synthesis in sow-reared piglets [15]. However, few studies have investigated the effects of NCG on mucosa-associated lymphatic tissue (MALT) function and intestinal IgA. We hypothesized that dietary NCG supplementation, which activates endogenous synthesis of arginine and increases serum arginine levels, could improve intestinal mucosa immunity after an *E. coli* challenge. Therefore, the objective of this study was to evaluate whether NCG supplementation could attenuate gut inflammation through stimulating gut-associated lymphoid tissu (GALT) functions and intestinal IgA response after *E. coli* K88 challenge in piglets.

## Materials and Methods

### Animals and Experimental Design

Twenty-eight 4-day-old male Landrace×Large White piglets were obtained from by a commercial pig farm and transported to the Laboratory of Animal Metabolism at China Agricultural University (Beijing, China). All procedures of this experiment complied with the animal care protocol which was approved by the China Agricultural University Animal Care and Use Committee. And China Agricultural University Animal Care and Use Committee specifically approved this study. NCG was purchased from Sigma-Aldrich Corporate (Louis, Missouri, US).

The piglets were assigned into four groups in a randomized complete block design according to their initial body weight: sham challenge (I), sham challenge + NCG (II), *E. coli* challenge (III), *E. coli* challenge + NCG (IV). Diets in group II and group IV were supplemented with 50 mg/kg body weight NCG added in Milk-replacer formula. *E. coli* was administered as a pathogen to establish the model of intestinal inflammation. Piglets were housed in individual metabolic cages (0.7 m×1.7 m) in a temperature controlled nursery room (32–34°C for the first week, 30–32°C for the second week ). Two sham challenge groups and two *E. coli* K88 challenge groups were housed in two separate nursery rooms. The composition and nutrient levels of the milk-replacer formula are shown in Table 1. The Milk-replacer formula was diluted to one-fifth of its concentration with drinking water on the basis of dry material concentration of sow’s milk. All the piglets were artificially fed every 4 hours using nursing bottles. Meanwhile, metal sheet were put under the nursing cages in order to collect the formula waste; therefore, the intake of formula was recorded accurately.

**Table 1:** Ingredient and chemical composition of the milk-replacer formula^1^.

Component	Milk-replacer
Crude Protein %	25.86
Energy MJ/kg^2^	20.28
Lactose %	34.80
Calcium %	0.95
Total Phosphorus %	0.75
The analyzed contents of amino acids in diets %	
Essential	2.60
Threoline	1.75
Valine	1.42
Isoleucine	1.40
Leucine	2.42
Phenylalanine	0.91
Histidine	0.50
Lysine	2.03
Nonessential and conditionally Essential	
Asparate	2.60
Tyrosine	0.77
Serine	1.32
Glutamate	4.38
Proline	1.46
Glycine	0.57
Alanine	1.25
Argine	0.75

1 A Milk-replacer formula (purchased from Dacheng, Taiwan). Diets were analyzed for crude protein, calcium, and phosphorus contents according to Association of Official Analytical Chemists (2003) procedures [34]. Dietary amino acids were determined by ion-exchange chromatography using Hitachi L-8800 Amino Acid Analyzer (Tokyo, Japan).

2 Based on milk-replacer.

On d 8, all the piglets were weighed again. Piglets in the *E. coli* challenged groups were orally administrated with 5 mL *E. coli* K88 (10^8^ CFU/mL, purchased from the Chinese Academy of Sciences), the dose was provided by using a 10 cm tube attached on a syringe based on the results of our preliminary experiment; piglets in sham challenge groups, however, were administrated on equal volume of drinking water. The culture of *E. coli* K88 was grown for 20 h in a Luria broth at 37°C using 0.1 mL of inoculum from stock. Then, cells were washed twice using PBS. Next, the culture was centrifuged for 15 min at 3,000×*g*. Supernatants were discarded and cells were re-suspended in PBS at concentration of 10^8 ^CFU/mL of *E. coli* K88 (calculated based on the optical density established by serial dilution before viable bacterial count), which was directly used for the oral challenge to piglets.

On day 13, all the piglets were weighed and euthanized after overnight fast. Jugular venous blood samples from each piglet (5 mL) were obtained 4 h after the last meal. The blood samples were centrifuged for 10 min at 3,000×*g* to obtain serum samples, which were immediately stored at −20°C until sample analysis. A 15 cm section of each intestinal segment (at the middle location), including duodenum, jejunum and ileum, was flushed gently with 0.9% physiological saline before obtaining the mucosa (10 cm) and the intestinal segment (5 cm).

### Fecal Consistency and Diarrhea Incidence

The occurrence of diarrhea for each piglet was observed and visually assessed every afternoon after the challenge. According to this method, a scores of 0 represents normal and firm feces; 1 represents possible slight diarrhea; 2 represents definitely unformed and moderately fluid feces; and 3 represents very watery and frothy diarrhea [16]. The total diarrhea score of each group was calculated each day. The occurrence of diarrhea was defined as maintaining fecal scores of 2 or 3 for two days consecutively. The diarrhea incidence was calculated in accordance with the following formula: diarrhea incidence (%) = number of piglets with diarrhea×diarrhea days/(number of piglets×5)×100% [16], [17].

### Analyses of Immunoglobulins in Serum and Intestine

Serum samples were assayed for the concentrations of amino acids and immunoglobulin (IgA, IgG, IgM). Serum free amino acids were analyzed using S-433D Amino Acid Analyser (Skam) as previous described. The concentration of serum AA was determined by ion-exchange chromatography with physiological fluid analysis conditions (S-433D AA Analyzer, Sykam, Germany). After the frozen serum samples were thawed at 4°C, the thawed samples were deproteinized by using 120 mg of salicylic acid in each millilitre of serum. After a 20 min ice bath, the reaction system was adjusted by adding lithium hydroxide solution (2 mol/L) for pH value and then centrifuged at 45,000×*g* (L-80 XP, Beckman) for 30 min. Supernatant was collected and then filtered a 0.1 µm filter. Serum immunoglobulin proteins (IgA, IgG, IgM) were measured with a swine ELISA kit (Cusabio Biotech Company, Wuhan, China), and the analysis procedures followed the manufacturer’s instructions.

Duodenum, jejunum, and ileum tissue were isolated and the contents were removed. The mucosa was scraped gently from the intestines using a glass slide. Then, it was immediately immersed into liquid nitrogen and then stored at −80°C until use. Mucosa samples (0.1 g) were mixed in 5 mL PBS supplemented with 1% protease inhibitor (Sigma-Aldrich Company, Louis, Missouri, US). Samples were homogenized, and the homogenates were ultracentrifuged for 10 min at 5,000×*g*. The SIgA levels in the supernatant were measured by using a swine ELISA kits (Cusabio Biotech Company, Wuhan, China), and were normalized for the weight of each intestinal segment.

### Analyses of Enzyme and Cytokine in Intestine

Samples from the jejunum and ileum were weighed to 1.0 g and placed in 10 ml phosphate buffered saline at 4°C. Samples were homogenized for 30 s and then centrifuged immediately for 10 min at 5,000×*g*. The obtained supernatants were immediately stored at −20°C until sample analysis. Levels of cytokines (IL-4, IL-10, IL-2 and INF-γ) in ileum were measured using ELISA kits (Cusabio Biotech Company, Wuhan, China).

### Histology and Immunohistochemistry

Samples from the ileum were fixed with 4% paraformaldehyde, and were dehydrated, cleared, and embedded in paraffin. All the samples were cut into 5 µm thickness and placed on glass slides. Then, the slides were deparaffinized and endogenous hydrogen peroxidase was blocked by a 3% solution of hydrogen peroxide. After that, the slides were heated for 10 minutes in a pressure cooker in the presence of citrate buffer (pH 6.0) and blocked for 20 minutes with PBS containing 10% goat serum. The slides were incubated with primary anti-CD4, anti-CD8 or anti-CD19 overnight at 4°C. The sections were then washed with PBS and co-incubated with a secondary antibody of peroxidase conjugated anti-IgG (1∶500 in PBS, v/v) for 30 minutes at 37°C. Slides were exposed in a DAB substrate reagent (ZSGB-BIO, Beijing, China), and counterstained with Mayer’s hematoxylin (ZSGB-BIO, Beijing, China). Images were visualized using a microscope (Olympus BX41; Olympus Optical, Tokyo, Japan).

### Statistical Analysis

The experiment was a 2×2 factorial arrangement with the NCG supplementation and *E. coli* challenge being the main factors. Data were expressed as least squares means and standard error of the mean (SEM). Differences among treatments were analyzed by ANOVA for a randomized complete block design using the General Linear Model procedures of the Statistical Analysis System (SAS Inst Inc., Cary, NC). Probability values less than 0.05 were regarded as significance.

## Results

### Performance

Table 2 shows the performance of piglets before and after the challenge. There was no difference in body weight among the four treatments at the beginning of the experiment, as well as on day 8 and day 13. In addition, average daily gain (ADG) and average daily feed intake (ADFI) were also not significantly different among four groups before the challenge (day 1–7). However, the ADG was significantly decreased after *E. coli* K88 challenge (*P_E.coli_*<0.05), compared with sham challenge groups. Although no significant ADG difference was observed in NCG-supplemented groups compared with control diet groups, there was a trend that NCG-supplementation alleviated the weight growth underdevelopment and increased the average daily gain after *E. coli* challenge (*P*_NCG_ *= *0.08). We also did a t-test on sham-challenge groups and *E. coli-*challenge groups separately. We found that there was no difference in both sham-challenged groups and *E. coli*-challenged groups.

**Table 2:** Effects of N-carbamylglutamate supplementation on the performance of pigs during pre- and post-challenge periods.

	*E.coli*		NCG		SEM^2^	*P*-value		
	Sham^1^	Challenge Sham^1^	Non-supplementedsupplemented	Supplemented		*E. coli*	NCG	Interaction
Body weight (kg)								
Day 0	2.0	2.2	2.1	2.1	NC	NC	NC	NC
Day 8	3.1	3.2	3.1	3.2	0.27	NC	0.86	NC
Day 13	4.1	3.9	3.9	4.0	0.39	0.37	0.60	0.57
Day 1–8 (Before *E.coli* K88 challenge )								
ADG (g)	150	155	149	156	3.80	NC	0.45	NC
ADFI (g)	138	143	140	142	4.01	NC	0.84	NC
Day 8–12 (After *E.coli* K88 challenge)								
ADG (g)	163	149	150	161	3.21	<0.05	0.08	0.2
ADFI (g)	221	212	222	211	24.34	0.37	0.24	0.38
Diarrhea Incidence^3^ (%)	0	34.4	18.8	15.6	NC	NC	NC	NC

ADFI, average daily feed intake; ADG, average daily gain; NC, not calculated.

1 challenged with water.

2 standard Error of Mean.

3 diarrhea incidence (%) = number of piglets with diarrhea×diarrhea days/(number of piglets×5) ×100% [16], [17].

Diarrhea incidence of *E. coli* challenged piglets was significantly increased, compared with that of sham challenged piglets (Table 2). However, diarrhea incidence of piglets fed the NCG supplemented diets decreased by 20.5% in comparison with piglets fed the control diet.

### Serum Amino Acid Concentrations

Serum concentrations of arginine, ornithine and citrulline were significantly increased in NCG supplemented piglets (*P*_NCG_<0.05), which confirmed that NCG could continuously promote the endogenous arginine synthesis. No changes in circulating levels of other amino acids were observed in non-NCG supplemented piglets (Table 3).

**Table 3:** Selected plasma amino acid concentrations in serum (nmol/mL).

Amino acid group	*E.coli*		NCG		SEM^2^	*P*-value		
	Sham^1^	Challenge	Non-supplemented	Supplemented		*E. coli*	NCG	Interaction
Arginine	166.79	159.51	153.56	171.34	3.24	0.36	<0.05	0.73
Ornithine	67.49	69.73	63.40	74.72	1.67	0.42	<0.05	0.82
Citrulline	152.34	147.47	132.32	167.38	4.55	0.67	<0.05	0.74
Proline	585.98	582.98	584.32	583.08	2.53	0.52	0.94	0.48
Glutamine	569.35	561.12	567.19	563.97	6.51	0.68	0.88	0.96
Glutamate	198.38	189.56	193.21	195.20	3.3	0.10	0.79	0.84
Threonine	516.75	518.98	517.65	517.87	1.01	0.54	0.83	0.53
Histidine	86.51	87.18	85.83	88.46	2.97	0.98	0.38	0.75
Isoleucine	134.11	133.49	133.81	132.78	1.03	0.54	0.98	0.35
Leucine	192.13	194.71	192.83	195.00	4.19	0.53	0.44	0.82
Methionine	81.67	81.28	80.31	82.65	2.93	0.89	0.42	0.82
Phenylalanine	90.54	89.20	90.24	89.50	3.08	0.66	0.80	0.58
Tryptophan	40.84	40.24	39.17	41.92	2.15	0.78	0.21	0.97
Lysine	337.18	341.25	338.53	340.32	3.10	0.66	0.91	0.23

1 challenged with water.

2 standard Error of Mean.

### Serum and Intestinal Immunoglobulins

To determine the impact of NCG supplementation on different subsets of immunoglobulins in *E. coli* challenged piglets, the levels of immunoglobulins, including IgA, IgG, IgM, were detected by ELISA. The result (Table 4) indicates that *E. coli* challenge contributed to an obvious increase in several immunoglobulins in both serum and intestine compared with sham challenge groups (*P_E.coli_*<0.05). In addition, levels of IgA, IgM and IgG in serum were not affected by NCG supplementation (*P*_NCG_>0.1). Furthermore, the SIgA level in ileum was significantly increased with NCG supplementation (*P*_NCG_<0.05). However, the SIgA levels in duodenum and jejunum were unaffected by NCG treatment.

**Table 4:** Different subsets of immunoglobulin level in serum and small intestine.

	*E.coli*		NCG		SEM^2^	*P*-value		
	Sham^1^	Challenge	Non-supplemented	Supplemented		*E. coli*	NCG	Interaction
Serum (ug/mL)								
IgA	186.91	272.85	226.50	236.10	8.77	<0.05	0.46	0.76
IgM	521.99	621.90	566.62	573.84	12.59	<0.05	0.66	0.60
IgG	1683.29	2193.42	2081.67	1787.06	100.55	<0.05	0.95	0.99
SIgA (ug/g)								
Duodenum	21.01	26.13	22.90	24.53	1.50	0.12	0.48	0.63
Jejunum	43.01	53.80	47.85	49.65	2.11	<0.05	0.63	0.68
Ileum	49.55	64.82	55.10	60.15	1.32	<0.05	<0.05	0.07

1 challenged with water.

2 standard Error of Mean.

### Ileal Homogenate Cytokine Analyses

We also determined whether NCG supplementation affected the levels of IFN-γ, IL-2, IL-4, and IL-10 in ileal homogenates. As shown in Table 5, levels of IFN-γ, IL-2, IL-4 and IL-10 in ileal homogenates increased significantly responding to the *E. coli* challenge compared with pigs with sham challenge (*P_E. coli_*<0.05). The level of IL-10 also increased in NCG supplemented piglets (*P*_NCG_ = 0.05). On the other hand, IFN-γ and IL-4 were unaffected by the supplementation of NCG (*P*_NCG_>0.05). NCG supplementation had no effects on the level of IL-2 in non-*E. coli* challenged + NCG piglets, but had effects on *E. coli* challenged + NCG piglets. The supplementation of NCG blunted the increase of IL-2 which was induced by *E. coli* challenge (*P_interaction_*<0.05). Thus, we had another *t*-test between the *E. coli* challenged piglets and *E. coli* challenge + NCG group. The results revealed that the level of IL-2 decreased significantly in *E. coli* + NCG piglets compared with *E. coli* challenged piglets (*P<0.05*).

**Table 5:** Cytokine levels in ileum homogenates (pg/mL).

	*E.coli*		NCG			SEM^2^	*P*-value		
	Sham^1^	Challenge	Non-supplemented		Supplemented		*E.coli*	NCG	Interaction^3^
IFN-r	53.82	92.97	71.52	72.21		1.448	<0.05	0.38	0.74
IL-2	18.45	27.04	22.50	22.35		0.312	<0.05	0.18	<0.05
IL-4	131.42	179.36	144.07	162.72		10.627	<0.05	0.51	0.69
IL-10	1508.42	2290.87	1722.62	2036.08		69.933	<0.05	0.05	0.12

1 challenged with water.

2 standard Error of Mean.

3 Interaction = main effect of E.coli×main effect of NCG.

#### CD4^+^, CD8^+^ and CD19^+^ lymphocytes in lamina propria of ileum

The percentages of CD4^+^ and CD8^+^ T lymphocytes and CD19^+^ in lamina propria of ileum were measured by Histology and Immunohistochemistry. As shown in Table 6, supplementation with NCG increased the percentage of CD4^+^ T lymphocytes significantly (P_NCG_<0.05) rather than CD8^+^. Although no significant difference was observed in percentage of CD19^+^ lymphocytes, there was an increasing trend (*P*_NCG_ = 0.072).

**Table 6:** Numbers of lamina propria lymphocyte subsets in different treatments (%).

	*E. coli*		NCG		SEM^2^	*P-value*		
	Sham^1^	Challenge	Non-supplemented	Supplemented		*E. coli*	NCG	Interaction
CD4^+^	22.92	23.56	20.73	25.22	1.13	0.78	<0.05	0.14
CD8^+^	14.07	16.24	14.23	16.09	0.98	0.29	0.33	0.96
CD19^+^	34.06	34.65	32.94	35.77	0.75	0.71	0.072	0.99

1 challenged with water.

2 standard Error of Mean.

## Discussion

Accumulated research has indicated that dietary arginine supplementation can enhance the growth of milk-fed young pigs [18], improve intestinal function [19], decrease expression of inflammatory cytokines, and reduce intestinal mucosal injury in different animal models [8], [20], [21]. For example, Zhu et al. have studied the effect of L-arginine on intestinal mucosal immune barrier function in weaned pigs after *Escherichia coli* LPS challenge [21]. However, it is still very important to study the effect of NCG on mucosal immunity of neonatal piglets for mainly two reasons: 1) weaned pigs, as an animal model, is quite different with neonatal piglets. For weaned piglets, the weaning transition involves complex physiological changes, and the endogenous synthesis of arginine could satisfy the arginine requirement in normal condition [22]. For neonatal piglet, the immunity system and digestive systems have not adequately developed, and the endogenous synthesis of arginine declined significantly after birth [9]. So the immunity response of these two animal models maybe different when supplemented with arginine or alternative substitutes of arginine; 2) although both of NCG and arginine supplementations could increase plasma arginine concentration, NCG has a longer half-life compared with arginine, which means that it could increase the endogenous arginine synthesis continuously. Also NCG could avoid the negative effect of chronic provision of chloride on mammalian health caused by supplementation of L-arginine-HCl [9], [23].

NCG has been reported as having no toxicity in animals when administered orally [24]; it increases plasma concentrations of arginine, enhances intestinal growth and the expression of heat shock protein-70 in weaned pigs [19]. However, no report has revealed the effects of NCG on intestinal mucosal barrier in neonatal piglets.

In the present experiment, *E. coli* challenged piglets were used as a model to reveal the role of NCG supplementation in intestinal mucosal immunity. Enterotoxigenic *E. coli* K88 not only colonizes in the small intestine, but also releases enterotoxins, which leads to epithelial cells secreting fluid into the lumen of the gut and causes diarrhea [25]. Our results demonstrated that *E. coli* challenge decreased performance and increased the diarrhea incidence of piglets during post-challenge, which is in accordance with previous research [16]; however, the feed intake in challenged piglets was not influenced by *E. coli* challenge, indicating that the growth inhibition was caused by factors other than feed intake, such as impaired intestinal integrity, or limited nutrient digestion and absorption, especially the absorption of essential amino acids. Additionally, prior to *E. coli* challenge, NCG supplementation had no effect on performance of neonatal pigs which is in contrast to previous studies [15]. The reason for this inconsistence might be that the level of arginine in the mild-replacer formula used in this study was already adequate for neonatal growth under normal conditions, but not under the *E. coli* challenge. After *E. coli* challenge, NCG alleviated growth depression in *E. coli* challenge group because of the increase in the endogenous synthesis of arginine [19], which could improve protein synthesis, abate protein catabolism, and recover intestinal functions under depressed situations as well [8]. Therefore, we suggested that 50 mg/kg NCG supplementation promoted the endogenous arginine concentration, which partially alleviated growth depression under *E. coli* challenge by improving protein synthesis, abating skeletal muscle protein catabolism and promoting the recovery of intestinal functions.

It is well-known that immunoglobulins levels in serum and small intestine are important indicators of immune response [27]. Our results indicated that the concentration of SIgA was improved by NCG supplementation in the ileum. Additionally, the IgA and IgM levels in serum, although had no significant increasing when supplemented with NCG, had a similar trend with SIgA, which confirmed the effects of NCG on SIgA producing, because of previous research on the strong positive correlation between serum IgA concentration and local intestinal production [26]. SIgA, is mainly produced by mature IgA-secreting B cells in lamina propria [27], has been reported as playing quite an important role in protecting the intestinal mucosal surfaces [28]; it binds pathogens and their toxins and prevents their attachment to the lumen surface and infections.

SIgA is based on the numbers of lymphocytes in the lamina propria, and some kinds of cytokines are related to the IgA regulation, so NCG’s function of increasing SIgA may be due to that arginine’s effects on promoting B cells and CD4^+^ cell counts and some cytokines involved in IgA-regulation. In order to test this hypothesis, we measured CD4^+^ cells (helper T cells), CD8^+^ cells (cytotoxic T cells) and CD19^+^ cells (B cells) concentrations in intestinal lamina propria by immunohistochemistry. The results demonstrated that NCG supplementation significantly increased the proliferation of CD4^+^ cells and trended to increase CD19^+^ cells. This is consistent with previous studies that arginine proliferated CD4^+^ cells and CD19^+^ cells [29]. The explanation for this is that arginine is an essential amino acid for maximum proliferative responses to T cell activation signals transduced via the TCR-CD3 complex [29]. B cells become the major source of IgA precursor cells by undergoing class switch recombination to IgA secreting cells, which are heavily dependent on some cytokines secreted by activated T cells, such as IL-10 [28]. Our results also revealed that the number of CD8^+^ was not affected by the increased level of arginine. This is inconsistent with the previous studies of Ochoa [20], who found L-arginine significantly increased the proportion of CD8^+^ cells [20]. This discrepancy may be due to our use of newborn piglets as our animal model, the absolute number of CD8^+^ cells was low at birth, and a significant increment was observed from the 19^th^ to the 41^st^ day of age [30]; So our results suggest that the supplementation of NCG could not accelerate the proliferation of CD8^+^ in neonatal pigs. In conclusion, the number of CD4^+^ and CD19^+^ in NCG supplementation groups was increased, compared with the no-NCG supplementation groups.

It is also well-known that the processes of B cells maturation into IgA-plasma cells and IgA synthesis are highly controlled by cytokines produced by T cells [29]. T cells produce cytokines that are either IgA-inhibitory, such as IFN-γ, IL-2, or IgA-stimulatory, such as IL-4, IL-5, IL-6 and IL-10 [31]. IFN-γ and IL-2, mainly produced by Th1 cells, have the ability to activate T lymphocytes; IL-4 and IL-10, mainly produced by Th2 cells, are quite important for SIgA synthesis; For example, IL-4 stimulates B cells undergoing class switch recombination to IgA secretory cells and IL-10 promotes conversion of SIgA B cells to mature SIgA-secreting plasma cells [28]. In addition, Th1 and Th2 cells can antagonize each other’s actions, IFN-γ secreted by Th1 cells can block the proliferation of Th2 cells, and high concentrations of IL-4 or IL-10 produced by Th2 can inhibit the generation of Th1 cells and Th1 cytokine production [32]. The results revealed that the level of IL-2 decreased significantly in *E. coli* + NCG piglets compared with *E. coli* challenged piglets; This finding is inconsistent with previous research reporting that arginine supplementation led to increase IL-2 production in vitro [33]. However, this is inconsistent because the absolute level of IL-2 accumulation is not only dependent on its production but also on its utilization, and the L-arginine, at the level in our experiment, may preferentially affect IL-2 utilization rather than its production in piglets [20], especially when the level of IL-2 had already been kept at a high level after *E. coli* challenge. Moreover, our results revealed that NCG supplementation promoted IL-10 production. Hence, it is equally possible that the increased level of IL-10 contributed to inhibitory effects on IL-2 production [32]. The results also showed that the levels of IL-4 and IL-10 were also significantly increased after the *E. coli* challenge, and the IL-10 concentration was further promoted by the NCG supplementation, which can stimulate SIgA secretion [28].

In conclusion, the present study indicates that NCG supplementation in milk-replacer formula is beneficial for promoting gut mucosal immunity after *E. coli* challenge in neonatal piglets, which might be associated with the increase in SIgA levels, proliferation of lymphocytes, as well as changes in cytokine concentrations.
